# Comparative effect of Antivet®, Oplasture®, and a prototype paste on color change and microhardness of simulated stained enamel: an in vitro study

**DOI:** 10.1038/s41405-025-00393-x

**Published:** 2025-12-30

**Authors:** Dina Fayez S. Diab, Esraa Esmeail H. Elhaddad

**Affiliations:** 1 Conservative Dentistry Department, Faculty of Dentistry, Egyptian Russian University, Cairo, Egypt; 2 Conservative Dentistry Department, Faculty of Dentistry, Misr University for Science and Technology, Cairo, Egypt

**Keywords:** Minimal intervention dentistry, Non-bonded restorations

## Abstract

**Background:**

Micro-abrasion enables conservative stain removal with tooth preservation, enhancing color and microhardness in line with minimally invasive dentistry principles. The aim of this in vitro study was to compare the effects of three micro-abrasion materials Antivet®, Oplasture®, and a prototype paste (37% phosphoric acid with Aluminum oxide powder) against an untreated control on the color stability and microhardness of simulated stained enamel surfaces.

**Methods and material:**

A total of 120 extracted, caries-free human teeth were utilized. The teeth were randomly allocated into four groups (*n* = 30). Group 1 (control, no treatment), Group 2 (Antivet®: 21% hydrofluoric acid (HF)), Group 3 (Oplasture®: 6.6% hydrochloric acid (HCl) with silicon carbide), and Group 4 (prototype paste: 37% phosphoric acid with aluminum oxide powder). Specimens were stained by immersion in coffee or cola solutions at 37 °C for 8 h daily over 5 days, with interim storage in artificial saliva, and further subdivided (*n* = 15) by staining type. Color analysis and microhardness testing were performed at baseline, post-staining, and post-treatment. Data were analyzed using the Shapiro-Wilk test, Levene’s test, one-way ANOVA, and Holm’s method for multiple comparisons.

**Results:**

Statistically significant differences in enamel color change (Δ*E*) were observed among micro-abrasion materials versus control (*p *< 0.001), with the prototype paste inducing the highest ΔE after staining. Only Antivet® showed solution-dependent Δ*E* differences (*p* = 0.035). Enamel microhardness decreased significantly post-staining, confirming demineralization. Oplasture® achieved the greatest microhardness recovery, while Antivet® and the prototype paste were moderately effective. The control group exhibited the highest microhardness loss, after cola exposure (*p* = 0.045), highlighting the protective effect of micro-abrasive treatments.

**Conclusions:**

All tested micro-abrasive materials effectively enhanced tooth color and enamel microhardness. Antivet® appears to be a viable alternative to hydrochloric acid**-**based products. The prototype paste emerged as a promising, cost-effective alternative for stain management.

## Introduction

Tooth discoloration represents a prevalent clinical challenge with significant implications for dental esthetics and patient-reported outcomes. Contemporary dentistry offers a spectrum of treatment modalities for enamel discoloration, categorized by their degree of invasiveness: non-invasive, micro-invasive, and macro-invasive. Among these approaches, microabrasion has emerged as a particularly valuable conservative treatment option, effectively addressing superficial enamel defects and discoloration while preserving tooth structure [1, 2].

The process of modern microabrasion treatment involves removing a layer of enamel tissue. To reduce the spread of the compounds and guarantee the safety of the process, the optimum microabrasion method has a low acid content and uses abrasive particles in a water-soluble mixture applied with a slowly rotating handpiece. Several microabrasion products have been employed, and depending on the acid content and application time, the depth abraded has been reported to range from 20 to 200 µm. Surface discolouration is eliminated by acid erosion and the polishing effect of abrasive particles [3].

The micro-abraded enamel surface undergoes structural modification, forming an aprismatic layer characterized by increased smoothness, compactness, and optical regularity. This polished surface exhibits enhanced light reflectance, contributing to the effective masking of underlying discoloration. Furthermore, the transformation from a prismatic to a more homogeneous structure yields a denser, highly mineralized surface layer, thereby improving enamel resistance [2, 4].

Initially employing strong acids like HCl, modern techniques now use safer formulations, such as phosphoric acid with abrasives, though concerns remain over enamel dissolution. Despite refinements, optimal protocols for efficacy and safety require further investigation [5, 6]. The recent introduction of Antivet® (21% hydrofluoric acid) proposes a novel approach based on the calcium-chelating mechanism of a weak acid, yet its comparative efficacy remains underexplored. Therefore, this study evaluates and compares the effects of three micro-abrasion materials Antivet®, Oplasture® and a prototype paste (37% phosphoric acid with Aluminum oxide powder) against an untreated control on the color stability and microhardness of simulated stained enamel surfaces. The null hypothesis posits no significant difference between Antivet®, Opalustre®, and a 37% phosphoric acid prototype paste on color change or microhardness.

## Subjects and methods

### Experimental design

The protocol was approved by the Ethics Committee of the Faculty of Dentistry, Ain Shams University (FDASU-RecER042506). A total of 120 caries-free extracted anterior teeth without cracks, fractures, stains, or hypoplastic defects on the buccal surfaces were selected in this study. The required sample size calculation was performed using R statistical analysis software version 4.4.1 for Windows. By adopting an alpha (*α*) level of 0.05, a beta (*β*) level of 0.2 (i.e., power = 80%), and an effect size (*f*) of 0.800, the total required sample size (n) was calculated to be 80 samples. The sample size was increased to 120 samples (i.e., 30 samples per group and 15 samples per subgroup) to account for possible variability and failures in testing. Teeth were preserved in saline solution with 0.1% thymol solution (an antimicrobial solution that inhibits bacterial growth) until the study started [4, 7].

### Sample preparation

The teeth were rinsed with tap water and scaled to remove organic and inorganic debris. Then, they were polished with non-fluoridated pumice slurry (Prophy paste) using one prophylaxis brush mounted to a low-speed handpiece [8]. The roots of the teeth were removed, and the crowns were cut into 5-mm blocks with a diamond disk on a low-speed cutting machine (Isomet 1000, Buehler, Lake Bluff, IL, USA). With their labial surfaces exposed, the specimens were embedded in transparent auto-polymerizing acrylic resin (Acrostone, Egypt). A 6 mm circular adhesive tape was placed on each tooth’s buccal surface, then all other surfaces were coated with acid-resistant nail polish. After removing the tape, this created a standardized circular test area on each buccal surface [9]. Subsequently, the specimens were rinsed under running deionized water for 3 min and stored in a moist environment at 4 °C in a refrigerator [10].

### Staining, treatment and grouping

Coffee and cola were used as staining solutions, and distilled water was used on the control subgroup. The coffee was made with two grams of Nescafe powder (Nestle Szerencs, Hungary) and 200 mL of hot water. The solutions were refreshed every day [1]. The specimens were maintained in a stirring staining solution at 37 °C for 8 h, with the temperature held constant using a thermostatically controlled water bath. The specimens were then rinsed, allowed to dry, and then immersed in artificial saliva to simulate the oral environment overnight for five successive days [7]. The artificial saliva was prepared at the Faculty of Pharmacy, at Misr University for Science and Technology. It was composed of 4200 mg/L NaHCO_3_, 3 mg/L NaCl and 200 mg/L KCL, and Potassium Hydroxide (KOH) was added to adjust the pH to 7.4 [11].

### Specimens were randomly divided into the following study groups

The composition of the materials used in the study is listed in Table 1

**Table 1:** Composition of the materials used in the study.

Name	Composition
**Antivet®**	The primary component is a 21% hydrofluoric acid stabilized by an organic tricarboxylic acid The second component is Antivet alkaline base (It is calcium hydroxide with a pH >12) (Antivet- Antivet base, Dental Continental, S.A. de C.V. Industria del Plástico 2113 Fracc. Zapopan Industrial Norte Zapopan Jalisco México)
**Opalustre®**	6.6% hydrochloric acid paste with silicone carbide fine particles (Opalustre, Ultradent Products Inc, South Jordan, UT, USA)
**Paste prototype**	37% phosphoric acid and 50 µm aluminum oxide powder.

Group 1: Received no treatment and was stored in distilled water.

Group 2: Treated with 21% hydrofluoric acid (Antivet® MDC).

Group 3: Treated with hydrochloric acid at 6.6% and silicon carbide particles (Opalustre® Ultradent).

Group 4: Treated with a prototype micro-abrasive paste composed of 37% phosphoric acid and 50 µm alpha aluminum oxide powder (α-Al₂O₃).

### Enamel micro-abrasion technique

#### Antivet ® enamel micro-abrasion

The Antivet® abrasive solution (MDC DENTAL, Zapopan Jalisco, México) containing 21% hydrofluoric acid was placed on the buccal surface with a cotton swab. When the swab became pigmented, it was changed to another clean swab with the abrasive solution. The procedure was repeated for 10 min. When the process was finished, the excess solution was cleaned using cotton, and calcium hydroxide as a neutralizing solution was placed for 1 min with a micro brush. In the end, the samples were rinsed with water for 30 s [12].

#### Opalustre® enamel micro-abrasion

A water-soluble gel (Opalustre®, Ultradent Products Inc., South Jordan, Utah, USA) containing mildly concentrated hydrochloric acid (6%) and a fine-grit silicon carbide abrasive paper was applied to the enamel surface using a rubber cup (OpalCup™, Ultradent) at 500 rpm for 30 to 40 s. The abrasive compound was applied in three applications of 1 min each, with irrigation between each application [13].

#### Prototype enamel micro-abrasion mix paste

A 37% phosphoric acid gel (VOCO GmbH, Germany) was used as the erosive agent, combined with 50 µm alpha aluminum oxide powder as the abrasive material to prepare a paste. The paste was mixed at a 1:1 volume ratio using a standardized dispensing spoon [14]. Micro-abrasion was performed in three applications of 1 min each, employing a contra-angle micromotor with a rubber cup (Opti 1 Step™, Kerr) at 500 rpm for 30–40 s per application. Subsequently, the teeth were rinsed with water for 30 s to remove residual material.

After micro abrasion treatment in different groups, fluoride varnish (Bifluorid 10 by VOCO, Germany). was applied as a remineralizing agent on the enamel surface for four minutes.

### Color assessment

Color assessment L*a*b* values (Commission Internationale de l’Eclairage) were assessed for each specimen at three experimental stages: baseline, post-staining, and following treatment application. Each color measurement was done using a calibrated spectrophotometer (VITA Easyshade^®^V, VITA Zahnfabrik, Germany), with each specimen air-dried for 30 s prior to measurement [15]. All color measurements were conducted under standardized conditions according to ISO/TR 28642-2016 [16]. To ensure measurement consistency, a single operator performed all assessments. Measurements were conducted under controlled conditions at midday in a standardized location to minimize variability due to external lighting. A neutral gray background was employed for all color evaluations, as it was determined to exert minimal influence on spectrophotometric readings [17]. The color difference (Δ*E*) was calculated at baseline (Δ*E*_Baseline_, after staining (Δ*E*_Staining_), and after the assigned treatments (Δ*E*_treatment_: treatment-staining): Δ*E* = {(ΔL*)^2^ + (Δa*)^2^ + (Δb*)2}^1*/*2.^

### Microhardness assessment

Vickers microhardness testing was conducted using a calibrated microhardness tester (Model HVS-50, Laizhou Huayin Testing Instrument Co., Ltd., China) equipped with a diamond indenter and 20× objective lens. For each specimen, three indentations were made with a 25 mg load applied perpendicular to the buccal surface for 15 s, maintaining an inter-indentation distance of 0.5 mm. The diagonal lengths of the indentations were automatically measured by the integrated micrometer and converted to Vickers hardness numbers (VHN). The mean value derived from these measurements represented the final hardness value for each specimen [7].

### Statistical analysis

Numerical data were represented as mean and standard deviation (SD) values. They were explored and confirmed for normality by viewing the distribution and using the Shapiro-Wilk test. ANOVA models were built to test the effect of different tested variables on measured outcomes. The models were tested for normality of residuals using the methods as mentioned earlier and for variance homogeneity using Levene’s test and linearity by visual inspection of residual vs fitted values. For color change models, all assumptions were validated; however, for the microhardness models, the assumptions were only valid after Yeo-Johnson transformation. All further statistical analyses for the microhardness data were performed on the transformed data, while the original data were used in descriptives for interpretability. Post-modal comparisons of simple main effects were made utilizing the error term of the models with p-value adjustment using Holm’s method. The significance level was set at p < 0.05 within all tests. Statistical analysis was performed with R statistical analysis software version 4.5.1 for Windows.

## Results

The statistical models evaluating the influence of various factors on color change demonstrated no significant variation among the tested solutions in terms of initial color change following staining. However, a significant interaction effect (*p* < 0.05) was identified between the micro-abrasion material composition and the staining solution, affecting both post-treatment color change and the total color change values. These findings are presented in Table 2.

**Table 2:** Color models.

Delta E	Parameter	Mean square	*f*-value	*p*-value	PES (95% CI)
**Baseline—Staining**	**Staining solution**	5.52	**0.38**	**0.540**	**0.005 (0.000 to 0.060)**
**Staining—Treatment**	**Material**	765.61	**62.54**	<0.001*	**0.743 (0.637 to 0.791)**
	**Staining solution**	41.96	**3.43**	**0.069**	**0.050 (0.000 to 0.155)**
	**Material** ^*^ **Solution**	36.62	**2.99**	0.037*	**0.121 (0.004 to 0.220)**
**Baseline—treatment**	**Material**	9.13	**0.79**	**0.502**	**0.032 (0.000 to 0.088)**
	**Staining solution**	240.33	**20.85**	<0.001*	**0.227 (0.096 to 0.353)**
	**Material** ^*^ **Solution**	43.17	**3.74**	0.015*	**0.137 (0.016 to 0.235)**

*PES* partial eta squared, *CI* confidence interval.

*significant (*p* < 0.05).

**Fig. 1:**
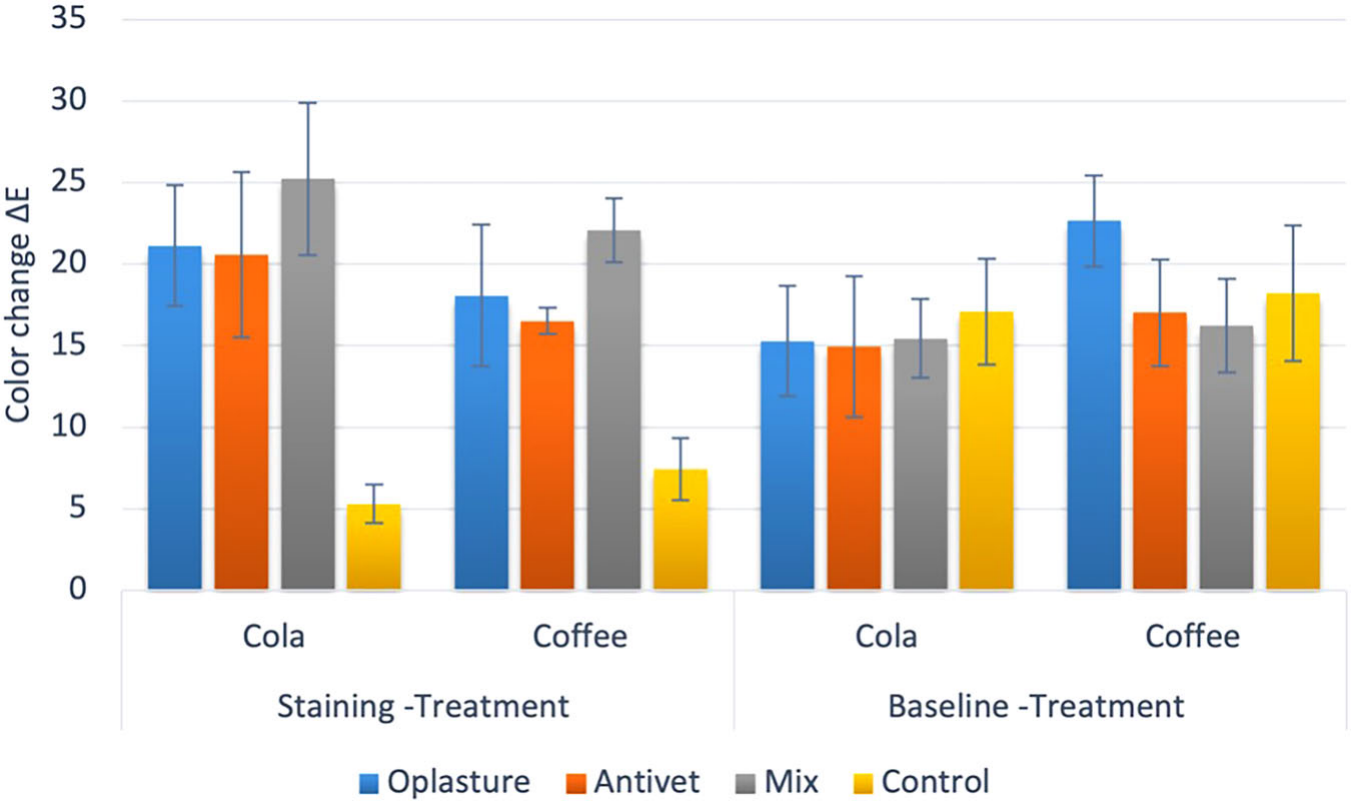
Bar chart showing mean and standard deviation values of color change.

Simple effects comparisons presented in Table 3 and Fig. 1 showed that, regardless of the staining solution, there was a significant difference in color change after treatment between different groups. Pairwise comparisons revealed that the prototype enamel microabrasion paste group had a significantly higher color change than the Antivet ®and control groups. Additionally, Antivet® had a significantly higher color change than the control group. For Antivet ®, the color change measured with cola was significantly higher than that with coffee. In contrast, the difference was not statistically significant for all other groups.

**Table 3:** Comparisons of simple main effects for color change.

*Delta E*	*Solution*	*Mean ± *SD				*p-*value
		Oplasture	Antivet	Mix	Control	
***Staining -Treatment***	**Cola**	21.13 ± 3.71^AB^	20.58 ± 5.09^B^	25.23 ± 4.69^A^	5.30 ± 1.16^C^	<0.001*
	**Coffee**	18.08 ± 4.35^AB^	16.52 ± 0.80^B^	22.08 ± 1.94^A^	7.42 ± 1.91^C^	<0.001*
	**p-value**	**0.069**	0.035*	**0.054**	**0.181**	
***Baseline -Treatment***	**Cola**	15.29 ± 3.38^A^	14.95 ± 4.34^A^	15.45 ± 2.43^A^	17.08 ± 3.25^A^	**0.502**
	**Coffee**	21.65 ± 2.79^A^	17.01 ± 3.26^B^	16.25 ± 2.87^B^	18.22 ± 4.18^AB^	0.007*
	**p-value**	<0.001*	**0.182**	**0.593**	**0.456**	

Values with *different superscripts* within the *same horizontal row* are significantly different; *significant (*p* < 0.05).

For the overall color change, there was no significant difference between the different groups immersed in cola. However, the difference was statistically significant for coffee samples, with the Oplasture® group having significantly higher change than the Antivet® and prototype enamel micro abrasion paste groups. For Oplasture®, the color change measured with coffee was significantly higher than with cola. In contrast, the differences between the two solutions were not statistically significant for the other groups.

Table 4 presents the models studying the effects of different variables on microhardness change. Results showed that the initial reduction in microhardness after staining, measured with cola (257.82 ± 40.23), was significantly higher than that of coffee (141.97 ± 81.92). Additionally, results revealed further changes in microhardness; there was a significant interaction between the type of staining solution and microabrasion material.

**Table 4:** Microhardness change models.

Microhardness change	Parameter	Mean square	f-value	p-value	PES (95% CI)
**Baseline – Staining (Reduction)**	**Staining solution**	515154.09	**32.90**	<0.001*	**0.471 (0.263 to 0.602)**
**Staining -Treatment (Increase)**	**Material**	28864.27	**4.48**	0.010*	**0.296 (0.049 to 0.431)**
	**Staining solution**	102378.58	**15.89**	<0.001*	**0.332 (0.117 to 0.497)**
	**Material** ^*^ **Solution**	20385.43	**3.16**	0.038*	**0.229 (0.008 to 0.366)**
**Baseline -treatment (Reduction)**	**Material**	2748.33	**3.06**	0.045*	**0.254 (0.003 to 0.397)**
	**Staining solution**	1068.11	**1.19**	**0.285**	**0.042 (0.000 to 0.206)**
	**Material** ^*^ **Solution**	2703.27	**3.01**	0.048*	**0.251 (0.002 to 0.394)**

*PES* partial eta squared, *CI* confidence interval.

*Significant (*p* < 0.05).

**Fig. 2:**
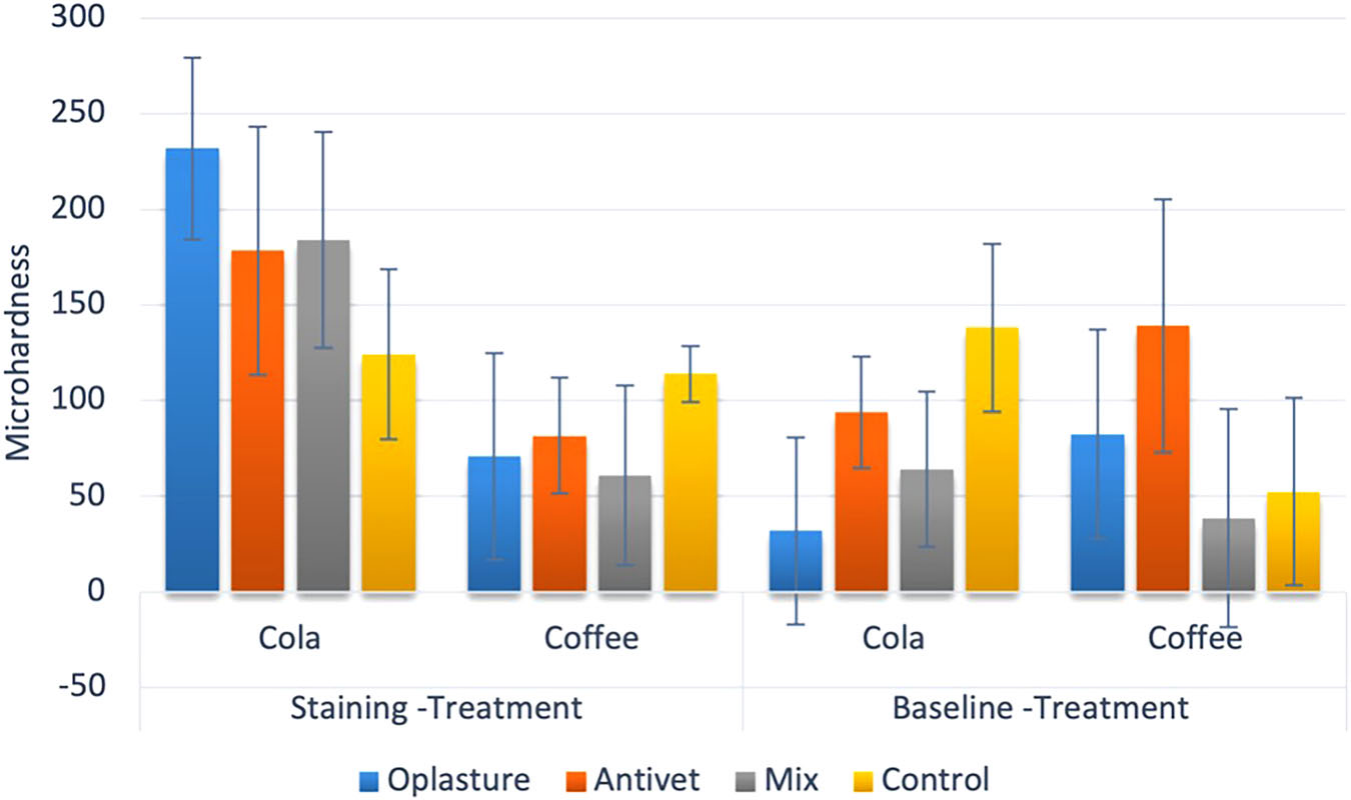
Bar chart showing mean and standard deviation values of microhardness change.

As shown in Table 5 and Fig. 2, the microhardness enhancement following microabrasion in cola-stained samples treated with Oplasture ® was significantly greater compared to other agents. In contrast, no statistically significant difference in microhardness increase was observed among coffee-stained samples. Additionally, with the exception of the control group, the microhardness improvement in cola-treated specimens was significantly higher than that in coffee-treated specimens.

**Table 5:** Comparisons of simple main effects for changes in microhardness.

*Microhardness change*	*Solution*	*Mean ± SD*				*p-value*
		Oplasture	Antivet	Mix (37% Phosphoric acid and Alumina oxide)	Control	
***Staining -Treatment (Increase)***	**Cola**	231.84 ± 47.59^A^	178.29 ± 65.15^AB^	184.01 ± 56.59^AB^	124.25 ± 44.27^B^	0.010*
	**Coffee**	70.78 ± 53.81^A^	81.59 ± 30.21^A^	60.96 ± 46.80^A^	113.89 ± 14.72^A^	**0.318**
	**p-value**	<0.001*	0.003*	<0.001*	**0.723**	
***Baseline -treatment (Reduction)***	**Cola**	32.05 ± 49.02^B^	93.71 ± 29.08^AB^	64.00 ± 40.41^AB^	138.14 ± 43.98^A^	0.045*
	**Coffee**	82.33 ± 54.73^A^	139.19 ± 66.16 ^A^	38.53 ± 56.80^A^	52.37 ± 48.98^A^	**0.097**
	**p-value**	0.285	0.127	0.938	0.028*	

Values with *different superscripts* within the *same horizontal row* are significantly different; *significant (*p* < 0.05).

In terms of the overall reduction of microhardness starting from baseline, for cola samples, the control group had significantly higher values than Oplasture®, while for coffee samples, the difference was not statistically significant. The reduction in the control group samples immersed in cola was significantly higher than that in the samples immersed in coffee. However, for other groups, the differences were not statistically significant.

## Discussion

Micro-abrasion is a cornerstone of minimally invasive dentistry, providing an effective, conservative method for managing enamel discoloration while preserving sound tooth structure [18]. This study provides a comparative analysis of three chemo-mechanical systems Antivet® (21% HF), Oplasture® (6.6% HCl), and a prototype (37% Phosphoric acid with Aluminum oxide powder) evaluating their efficacy in restoring enamel color and microhardness. The results demonstrated that all tested materials significantly improved both parameters, confirming the procedure’s overall efficacy. However, the extent of improvement varied according to each material’s specific chemical and mechanical composition, highlighting the critical interplay between acid strength and abrasive characteristics. These findings refine the current understanding of enamel micro-abrasion by identifying material-specific performance patterns that parallel, and in some aspects surpass, those reported in previous studies [3, 6, 19].

The deliberate use of coffee and cola provided a critical model to dissect these material-specific actions. Coffee, rich in tannins and polyphenols, creates deep, protein-bound chromogenic complexes, whereas cola’s staining is primarily driven by acidic, caramel-based colorants that pigmented a demineralized surface [20, 21]. This dual model allowed differentiation between pigment-based and acid-based staining responses and enabled an assessment of the material-specific effectiveness of the three systems. While the primary clinical application of micro-abrasion often targets developmental enamel defects, the use of severe extrinsic stains in this model served as a standardized challenge to effectively compare the fundamental stain-removal efficacy and enamel interaction of the different chemical-abrasive system.

Color change assessment in the current investigation was conducted using a standardized (VITA Easyshade) spectrophotometer, a device with well-documented sensitivity, reliability, and accuracy in dental color measurement [3, 16]. Recent advancements in optical electronics and digital technology have enhanced the accessibility and clinical applicability of such precise color evaluation systems. The instrument utilizes the (CIE)L*a*b* color space system, which offers objective quantification of chromatic parameters and facilitates longitudinal monitoring of color alterations.

The induced enamel staining resulted in a mean Δ*E* exceeding 3.3, confirming a visually detectable color change [10, 22]. Analysis of the treatment effects revealed that the prototype paste (37% phosphoric acid with aluminum oxide powder) induced the most profound color alteration, with post-treatment Δ*E* values of 25.23 ± 4.69 for cola and 22.08 ± 1.94 for coffee. This represents a stark contrast to the minimal changes observed in the control group (Δ*E* = 5.30 ± 1.16 and 7.42 ± 1.91).

The superior color change achieved with the prototype paste supports prior observations that phosphoric acid produces controlled surface etching with minimal subsurface damage as mentioned by Pini et al. [14]. Unlike hydrochloric or hydrofluoric acids, which induce rapid, non-selective demineralization, phosphoric acid appears to create a smoother and more uniform surface that optimizes light reflection without excessive tissue loss [19, 23] This aligns with Da Silva et al. [6], who emphasized that micro-abrasive efficacy depends not only on chemical dissolution but also on the creation of an optically homogeneous, compact enamel layer [3].

Aluminum oxide (Al₂O₃) has been established as the preferred abrasive material for enamel microabrasion, with well-documented clinical effectiveness in addressing both extrinsic stains and developmental enamel defects [24, 25]. Its particle morphology allows selective removal of weakened, pigmented enamel regions while preserving sound structure, a process enhanced by the 37% phosphoric acid matrix that facilitates gentle etching and easy manipulation. This synergy explains the prototype paste’s superior color recovery and supports its potential as a cost-effective alternative for clinical practice, particularly in resource-limited environments.

Moreover, Antivet® demonstrated a significantly greater color change compared to the control group. This effect may be explained by the behavior of hydrofluoric acid (HF), a weak acid that undergoes incomplete ionization in aqueous solutions. In acidic environments, such as enamel, HF exists in an equilibrium between its dissociated (H⁺ and F⁻) and undissociated (HF) forms. Due to this partial ionization, HF exhibits enhanced diffusion and penetration into the enamel, despite its weak acidic nature [2]. Consequently, this property facilitates more effective stain removal and a more pronounced color change.

The superior stain removal efficacy of Antivet® on cola stains, compared to coffee, can be mechanistically explained by the synergistic interaction between hydrofluoric acid (HF) and the stain’s composition. Cola’s primary pigments are phosphoric acid-based caramel colorants, which adhere via superficial demineralization. HF, despite being a weak acid, possesses a unique ability to penetrate enamel due to its low dissociation constant [17]. This allows it to effectively chelate and dissolve the mineral phase that has incorporated these cola pigments. Furthermore, the formation of soluble calcium-fluoride complexes (CaF₂) facilitates the removal of the stained layer without aggressive erosion [17], making it particularly effective for this type of stain. In contrast, coffee stains consist of robust tannins and polyphenols that form strong, hydrophobic complexes with enamel proteins [21, 26]. The chemical action of HF appears less effective at disrupting this protein-stain matrix compared to the strong, rapid demineralization caused by HCl in Opalustre®.

In contrast, for Oplasture®, the color change observed with coffee was significantly greater than that with cola. This difference is attributable to coffee stains, which primarily consist of tannins and polyphenolic compounds capable of binding strongly to enamel proteins through hydrogen bonding and hydrophobic interactions [21, 26]. Hydrochloric acid (HCl), as a strong mineral acid, induces rapid and profound demineralization of enamel, effectively disrupting the protein–stain matrix [27]. Consequently, HCl facilitates the superior removal of deeply embedded coffee pigments.

The divergent performance between the three micro-abrasion systems underscores the critical interplay between chemical and mechanical actions, a difference attributable to variations in their particle size, hardness, and compositional formulation [28, 29]. Opalustre’s® combination of strong HCl and silicon carbide likely provides a potent chemo-mechanical action, explaining its superior performance on demineralized surface where stain and compromised enamel must be removed. This aligns with studies by Rodrigues et al. [19], which emphasize the role of aggressive demineralization in stain removal [19]. In contrast, the prototype paste’s high ΔE suggests its efficacy is driven more by the potent abrasive action of 50 μm aluminum oxide, efficiently abrading the stained surface, as noted by Costa et al. [24, 30]. Antivet® presents a middle ground; its HF chemistry offers a selective, penetrating etch rather than a surface-wide demineralization. This novel mechanism, less documented in the micro-abrasion literature compared to HCl or phosphoric acid systems, may explain its balanced performance and suggests a potentially wider safety margin, a hypothesis that warrants further investigation into enamel loss [12].

Microhardness testing is regarded as an efficient and reliable approach for deriving indirect measurements of mineral content alterations in dental hard tissues [7]. The current study evaluated the effects of various micro-abrasive treatment on the microhardness of enamel following exposure to staining solutions. The results revealed significant interactions between treatment material and staining solution in determining enamel microhardness recovery and loss, indicating that both the type of abrasive system and the staining challenge play critical roles in the enamel’s mechanical response post-treatment.

The finding that Oplasture® facilitated the greatest recovery of microhardness (231.84 ± 47.59) after cola exposure is clinically significant, particularly given the substantial enamel-softening effect of staining agents confirmed by the large effect size (PES = 0.471). This suggests that its treatment not only removes the stain but also effectively eliminates the underlying softened, demineralized enamel layer, leaving a harder, more resilient surface. This is a crucial advantage when treating stains caused by acidic beverages, as it concurrently addresses the esthetic concern and the structural defect. However, a critical consideration not captured in this in vitro study is the potential for greater absolute enamel loss with such an aggressive agent. While our study measured the net improvement in surface hardness, clinical translation requires balancing this efficacy with tissue preservation. This concern is supported by the work of Da Silva et al. [6], which highlights the more aggressive enamel dissolution associated with stronger acids like HCl, underscoring that material selection must be guided by a balance of efficacy and conservation [3].

The difficulty in recovering microhardness after coffee staining, as observed in our study, resonates with the work of Carlos et al. [20], who suggested that coffee pigments form deep, protein-based complexes that are resistant to purely surface-level abrasive interventions [20].

Regarding the reduction in microhardness from baseline to post-treatment, significant differences were observed for the cola group (*p* = 0.045), with the control showing the greatest reduction (138.14 ± 43.98) compared to treated groups, reinforcing the protective effect of micro-abrasive materials. The partial eta squared values for material effects (PES ranging from 0.254 to 0.296) indicate moderate effect sizes, emphasizing the clinical significance of material selection in preserving enamel hardness.

Interestingly, while cola-induced softening was effectively counteracted by micro-abrasion, the results for coffee-stained enamel were less consistent. None of the treatments led to significant microhardness reduction compared to the baseline for coffee-stained samples (p = 0.097), suggesting a lower erosive potential of coffee or different stain penetration mechanisms less responsive to abrasive removal [27, 31].

These results are consistent with previous studies [8, 25] showing that microabrasion can effectively remove surface stains and enhance enamel smoothness and mineral content, leading to improved microhardness. However, differences in outcomes based on the material used and the type of staining agent highlight the importance of selecting appropriate micro-abrasion protocols for each clinical situation, especially when treating lesions caused by highly acidic drinks such as cola.

Collectively, these findings extend current evidence on enamel micro-abrasion by demonstrating that chemical composition and particle morphology critically modulate both esthetic and mechanical outcomes. Oplasture® remains the gold standard for heavily demineralized surfaces, but Antivet® introduces a novel hydrofluoric-acid-based system that achieves clinically comparable outcomes with potentially reduced enamel loss. The prototype paste, through its purely mechanical action, represents a promising low-cost formulation for resource-limited settings. Future in vivo studies should quantify enamel loss, surface morphology, and long-term color stability to establish optimized, evidence-based clinical protocols.

Despite the controlled conditions, this in vitro study has several limitations. The static staining model, while standardized, cannot fully replicate the complex dynamics of the oral environment, including the protective role of the pellicle and the cyclical nature of demineralization and remineralization. Furthermore, the assessment was short-term; long-term studies are needed to evaluate the color stability and enamel surface wear over time. Future research should focus on clinical or in situ studies to validate these findings in a clinical context.

## Conclusions

Within the limitations of this in vitro study, the following conclusions can be drawn:

1. The prototype paste demonstrated superior stain-removal efficacy, as evidenced by the most profound color change (Δ*E*). Its composition of widely available and economical components establishes it as a highly effective and cost-efficient alternative for managing enamel discoloration.
2. Antivet® proved to be a viable and reliable micro-abrasion agent. Its unique hydrofluoric acid-based chemistry provided effective, stain-specific removal and consistent microhardness recovery, positioning it as a balanced and potentially more conservative alternative.
3. Opalustre® exhibited the most pronounced restorative effect on enamel microhardness, particularly following acidic demineralization by cola. This confirms its role as the most effective agent for scenarios where addressing structural compromise and restoring surface hardness is the primary clinical objective, though its potential for greater tissue loss warrants caution.

## Data Availability

The data that support the findings of this study are available from the corresponding author upon reasonable request.
